# miRNAs标志物对晚期肺鳞癌的预测价值

**DOI:** 10.3779/j.issn.1009-3419.2025.102.16

**Published:** 2025-05-20

**Authors:** Anna WANG, Jingjing CONG, Yingjia WANG, Xin’ge LI, Junjian PI, Kaijing LIU, Hongjie ZHANG, Xiaoyan YAN, Hongmei LI

**Affiliations:** ^1^266000 青岛，青岛大学附属医院肿瘤科; ^1^Department of Oncology, The Affiliated Hospital of Qingdao University, Qingdao 266000, China; ^2^250117 济南，山东第一医科大学基础医学院; ^2^College of Basic Medical Sciences, Shandong First Medical University, Jinan 250117, China; ^3^570000 海口，海南科技职业大学医药学院; ^3^College of Medicine, Hainan Vocational University of Science and Technology, Haikou 570000, China; ^4^266071 青岛，青岛大学青岛医学院; ^4^Qingdao Medical College, Qingdao University, Qingdao 266071, China

**Keywords:** 肺肿瘤, 晚期肺鳞癌, miRNAs, 生物标志物, 富集分析, Lung neoplasms, Advanced lung squamous cell carcinoma, miRNAs, Biomarkers, Enrichment analysis

## Abstract

**背景与目的:**

肺癌是全球癌症死亡的主要原因之一，约80%的肺癌属于非小细胞肺癌（non-small cell lung cancer, NSCLC），其中肺鳞癌（lung squamous cell carcinoma, LUSC）在NSCLC中占据重要比例。尽管肿瘤的综合治疗极大提升了患者的总生存期，但晚期LUSC患者的预后较差。急需一种生物标志物来预测晚期LUSC患者的预后，以便通过早期诊断，改善预后。研究发现miRNAs在肺癌组织中差异表达，并作为潜在的致癌或抑癌基因发挥作用，本研究旨在筛选出早期和晚期LUSC差异表达的miRNAs，构建用于预测晚期LUSC患者预后的一组miRNAs标志物。

**方法:**

从癌症基因组图谱（The Cancer Genome Atlas, TCGA）数据库中下载LUSC患者临床信息及miRNAs的相关数据。应用生物信息学方法分析数据，绘制受试者工作特征（receiver operating characteristic, ROC）曲线，利用多种在线分析工具预测靶基因，分析靶基因的潜在生物学机制。

**结果:**

两组间共鉴定出58个差异表达的miRNAs。根据LASSO回归筛选出7个miRNAs拟构建miRNAs标志物，又根据每个miRNAs的ROC曲线下面积（area under the curve, AUC）值，最终选取其中的4个mRNAs（miR-377-3p、miR-4779、miR-6803-5p、miR-3960）作为预测晚期LUSC患者的生物标志物。4个miRNAs联合的AUC值为0.865。富集分析显示这些靶基因富集在癌症通路、促分裂素原活化蛋白激酶（mitogen-activated protein kinase, MAPK）通路、丝氨酸/苏氨酸激酶（serine/threonine kinase, STK）及酪氨酸激酶信号通路等多种通路。

**结论:**

miR-377-3p、miR-4779、miR-6803-5p、miR-3960联合预测晚期LUSC患者预后能力良好，AUC可达0.865。

## Abstract (en)

**Background and objective:**

Lung cancer is one of the leading causes of cancer-related mortality worldwide, with above 80% of cases be non-small cell lung cancer (NSCLC), among which lung squamous cell carcinoma (LUSC) occupies a significant proportion. Although comprehensive cancer therapies have considerably improved the overall survival of patients, patients with advanced LUSC have a poorer prognosis. Therefore, there is a need for a biomarker to predict the progress of advanced LUSC in order to improve prognosis through early diagnosis. Previous studies have shown that miRNAs are differentially expressed in lung cancer tissues and play roles as potential oncogenes or tumor suppressors. The aim of this study is to identify differentially expressed miRNAs between early-stage and advanced-stage LUSC, and to establish a set of miRNAs that can predict the progress of advanced LUSC.

**Methods:**

Clinical data and miRNA-related data of LUSC patients were downloaded from The Cancer Genome Atlas (TCGA) database. Bioinformatic methods were applied to analyze the data. Receiver operating characteristic (ROC) curves were plotted, and various online tools were used to predict target genes, with subsequent analysis of the potential biological mechanisms of these genes.

**Results:**

A total of 58 differentially expressed miRNAs were identified between the experiment group and the control group. Seven miRNAs were selected for potential construction of a miRNA biomarker through LASSO regression, and based on the area under the curve (AUC) values of each miRNA, four of these miRNAs (miR-377-3p, miR-4779, miR-6803-5p, miR-3960) were ultimately chosen as biomarkers for predicting advanced LUSC. The AUC under the ROC curve for the combined four miRNAs was 0.865. Enrichment analysis showed that these target genes were involved in several pathways, including cancer-related pathways, mitogen-activated protein kinase (MAPK) signaling pathway, serine/threonine kinase, and tyrosine kinase signaling pathways.

**Conclusion:**

The combined use of miR-377-3p, miR-4779, miR-6803-5p and miR-3960 provides a good predictive ability for the progress of advanced LUSC patients, with an AUC of 0.865.

微小RNAs（microRNAs, miRNAs）是一类具有调控功能、长度为19到24个核苷酸不等的非编码单链小RNAs，成熟的miRNAs产物是由较长的初级miRNAs（pri-miRNAs）转录物通过核糖核酸酶连续加工产生的^[1]^。它通常与靶信使RNA结合，在转录后水平调控基因表达^[2]^，参与包括发育、代谢、转移和免疫应答等在内的多种生物学过程^[2]^。人类癌症中发现的第一个miRNA来自于慢性淋巴细胞白血病的研究^[3]^。有证据^[3]^表明，包括非小细胞肺癌（non-small cell lung cancer, NSCLC）在内的多种癌症中含有大量失调的miRNAs，并作为潜在的致癌基因或抑癌基因发挥关键生物学效应。分析这些miRNAs将极大促进癌症的早期诊断，提高患者的总生存期（overall survival, OS）。此外，miRNAs存在于癌症患者的循环核酸中，且在血浆中具有组织特异性和高度稳定性，并且血液样本易于获取且经济适用，这表明检测循环miRNAs可能是一种有效的非侵入性癌症诊断方法^[1,4]^ 。

肺癌是全球癌症死亡率最高的癌种^[4]^，其中NSCLC在肺癌中约占80%，而肺鳞癌（lung squamous cell carcinoma, LUSC）是NSCLC的主要亚型之一，尤其以吸烟的男性患者更为常见^[5]^。虽然癌症治疗有重大突破，免疫检查点抑制剂和靶向治疗的发展极大延长了患者的生存时间^[6]^，但晚期肺癌仍具有较高的患病率和死亡率，手术仍是目前治疗肺癌的主要手段。早期和部分可手术治疗的IIIA期LUSC患者的5年生存率为20%-80%，而IV期LUSC患者的5年生存率仅为5%，但大多数LUSC患者确诊时已处于晚期，错过最佳治疗时机^[7]^，疾病相关死亡率高，因此，有必要寻找一种标志物来预测晚期LUSC患者的预后，通过早期诊断，改善肺癌患者的OS^[4]^，提高患者的生存质量。

癌症基因组图谱（The Cancer Genome Atlas, TCGA），旨在通过大规模基因组测序和数据分析，揭示癌症的分子机制，推动个性化癌症治疗和癌症研究。本研究的目的是通过TCGA数据库中LUSC的miRNAs的数据，筛选早期与晚期LUSC之间差异表达的miRNAs，并构建一组具有高特异性和敏感性的miRNAs生物标志物，用于预测晚期LUSC患者预后，并通过相关线上分析平台，进一步预测miRNAs靶基因及相关信号通路，为进一步探讨机制研究提供参考价值，并为LUSC的诊断和治疗提供了潜在的有效指标。

## 1 资料与方法

### 1.1 数据下载及处理

从TCGA公共数据库中下载LUSC患者相关的miRNAs数据信息和患者的临床信息（https://portal.gdc.cancer.gov/），最终筛选出患者478例，其中I-IIIA期可切除患者456例，IIIB期不可切除和IV期患者22例。纳入标准：（1）具有明确的肿瘤原发灶-淋巴结-转移（tumor-node-metastasis, TNM）分期；（2）既往未接受过抗肿瘤治疗。排除标准：（1）TNM分期不明确；（2）既往接受抗肿瘤治疗。按照纳入与排除标准，按照1:1.5的比例，从I-IIIA期患者中随机抽取33例患者，作为对照组；IIIB-IV期患者共22例，作为实验组。

### 1.2 差异表达miRNAs的筛选和miRNAs标志物的构建 

使用R语言（4.3.2版本）和SPSS（26.0版本）进行数据分析并绘图。在R语言中应用“edgeR”对对照组和实验组的miRNAs表达数据进行差异分析，计算每个miRNA的倍数变化（fold change, FC），按照|log2 FC|>1和错误发现率（false discovery rate, FDR）<0.05的标准，筛选差异表达的miRNAs并得出上调和下调的miRNAs，通过LASSO回归，从差异表达的miRNAs中进一步筛选一组miRNAs。通过SPSS软件，运用*Logistic*回归和受试者工作特征（receiver operating characteristic, ROC）曲线绘制图像。得到一组用于预测晚期LUSC预后的miRNAs标志物。

### 1.3 靶基因预测及富集分析

使用TargetScan和miRWalk数据库预测每个miRNA的靶基因，并利用韦恩（Venn）图筛选交集靶基因。然后应用微生信等线上分析平台对获取的交集靶基因进行基因本体（Gene Ontology, GO）和京都基因和基因组百科全书（Kyoto Encyclopedia of Genes and Genomes, KEGG）富集分析。

### 1.4 构建蛋白质相互作用（protein-protein interaction, PPI）网络并筛选核心基因

应用String数据库绘制PPI网络，并应用Cytoscape软件的cytoHubba插件筛选潜在的关键基因，并构建核心基因PPI网络，对其进行可视化。并利用微生信等线上分析平台制作miRNAs与靶基因的网络，进行网络可视化。

### 1.5 统计学方法

采用R（4.3.2版）软件处理数据及生成图像。分类变量使用计数（*n*）和比例（%）表示，采用χ^2^检验进行比较。*LASSO*回归用于筛选miRNAs。SPSS软件绘制ROC曲线用来评估miRNAs标志物的预测能力。*P*<0.05表示差异具有统计学意义。

## 2 结果

### 2.1 患者的基线特征

从TCGA数据库中下载共478例LUSC患者的miRNAs数据信息，根据入组标准，共230例LUSC患者纳入研究，并通过随机抽样，最终确定两组样本数目，其中对照组33例、实验组22例。每个患者作为一个独立样本，检测出共2213种miRNAs，筛选去掉表达量低（表达量<0.5）的miRNAs，最终入组732个miRNAs，并根据每种miRNAs测序数据的每百万读数（reads per million, RPM）值，进行数据分析。患者临床信息见表1，两组之间的年龄和性别差异不具有统计学意义。

**表 1:** 研究样本临床特征

Characteristics		Control group (n=33)	Experiment group (n=22)	P
Age (yr)	>65	25 (75.7%)	15 (68.7%)	0.570
	≤65	8 (24.3%)	7 (31.3%)	
Gender	Male	15 (45.4%)	21 (95.4%)	0.132
	Female	18 (54.6%)	1 (4.6%)	
T stage	T1-2	25 (75.7%)	5 (22.7%)	<0.001
	T3-4	8 (24.3%)	17 (77.3%)	
N stage	N0-1	31 (93.9%)	4 (18.1%)	<0.001
	N2-3	2 (6.1%)	18 (81.9%)	

### 2.2 筛选差异表达的miRNAs

应用R软件中“edgeR”包分析对照组和实验组的miRNAs的差异性表达，共鉴定出58个差异表达的miRNAs，分析出上调的miRNAs有4个，下调的miRNAs有54个（图1）。

**图 1:**
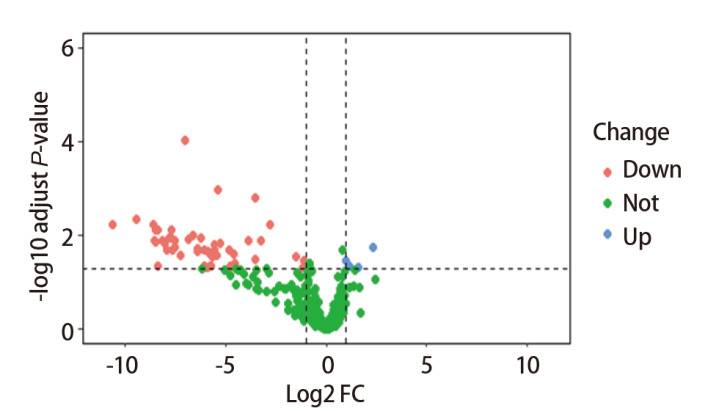
差异表达的miRNAs的火山图

### 2.3 初步构建miRNAs标志物

对58个miRNAs进行*LASSO*回归分析，根据回归分析所得曲线下面积（area under the curve, AUC）最大值，鉴定出7个miRNAs用于构建miRNAs标志物。这7个miRNAs包括miR-1914-5p、miR-377-3p、miR-580-5p、miR-4779、miR-3960、miR-6803-5p及miR-10a-5p（图2）。

**图 2:**
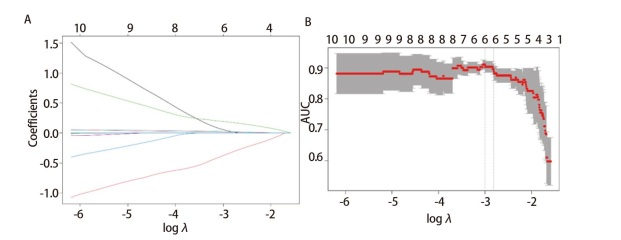
miRNAs标志物的构建。A：在LASSO分析中通过10倍交叉验证选择参数λ；B：7个差异表达miRNAs的LASSO回归系数谱。

### 2.4 miRNAs标志物对晚期LUSC的预测价值

通过SPSS软件绘制ROC曲线（图3）评估miRNAs标志物对晚期LUSC的预测价值，7个miRNAs标志物的AUC为0.960（95%CI: 0.914-1.000, P<0.0001），7个miRNAs的AUC值分别为：miR-10a-5p为0.339（95%CI: 0.194-0.482），miR-377-3p为0.664（95%CI: 0.513-0.815），miR-580-5p为0.256（95%CI: 0.126-0.385），miR-1914-5p为0.433（95%CI: 0.281-0.584），miR-3960为0.579（95%CI: 0.416-0.741），miR-4779为0.696（95%CI: 0.542-0.849），miR-6803-5p为0.686（95%CI: 0.537-0.835），但由于miR-1914-5p、miR-580-5p和miR-10a-5p的AUC值均小于0.5，不具备应用价值，因此，针对其他4个miRNAs进一步绘制ROC曲线进行分析，4个miRNAs联合的AUC值可达0.865（95%CI: 0.763-0.967, P<0.0001），这表明4个miRNAs的组合仍具有区分晚期LUSC患者的价值。因此最终选取4个miRNAs（miR-377-3p、miR-4779、miR-3960、miR-6803-5p）作为预测晚期LUSC的标志物。

**图 3:**
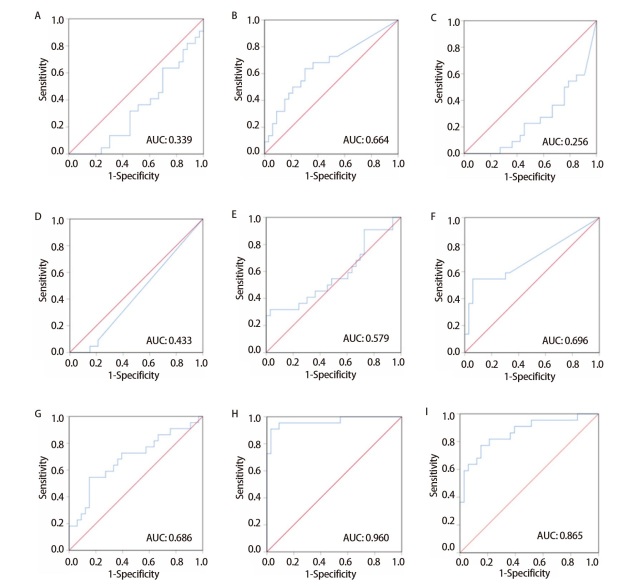
miRNAs标志物的预测价值。A：miR-10a-5p；B：miR-377-3p；C：miR-580-5p；D：miR-1914-5p；E：miR-3960；F：miR-4779；G：miR-6803-5p；H：7个miRNAs联合；I：4个miRNAs（miR-377-3p+miR-4779+miR-6803-5p+miR-3960）联合。

### 2.5 靶基因预测及富集分析

利用miRWalk和TargetScan数据库预测上述4个miRNAs的靶基因。运用韦恩图获取两个数据库中的交集靶基因，提高预测靶基因的精确性。miR-377-3p有42个重叠靶基因，miR-3960有99个重叠靶基因，miR-4779有968个重叠靶基因，miR-6803-5p有646个重叠靶基因（图4）。4个miRNAs共有1755个靶基因。然后，对1755个靶基因进行富集分析，以明确靶基因的生物学功能。

**图 4:**
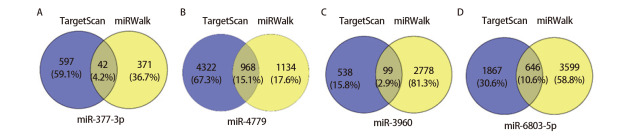
4个miRNAs重叠靶基因的韦恩图。A：miR-377-3p；B：miR-4779；C：miR-3960；D：miR-6803-5p。

借助微生信平台对1755个预测靶基因进行GO功能注释和KEGG通路富集分析。GO分析结果显示（图5），差异表达的靶基因在生物学过程（biological process, BP）中富集在神经递质水平的调节、神经递质转运、跨突触信号的调节等相关过程；在细胞成分（cellular component, CC）分析结果表明，这些靶基因富集在谷氨酸能突触、神经肌肉接头、神经元对神经元突触等过程；分子功能（molecular function, MF）分析显示，靶基因富集在GTPase活化蛋白结合、磷脂酰肌醇结合、磷脂酰肌醇二磷酸结合、STK活性、蛋白酪氨酸激酶活化等过程。KEGG途径（图6）集中在哺乳动物雷帕霉素靶蛋白（mammalian target of rapamycin, mTOR）信号通路、促分裂素原活化蛋白激酶（mitogen-activated protein kinase, MAPK）信号通路、癌症中的蛋白聚糖等多种与癌症相关的途径中。这表明我们筛选的miRNAs在LUSC的发展过程中发挥了潜在的作用。

**图 5:**
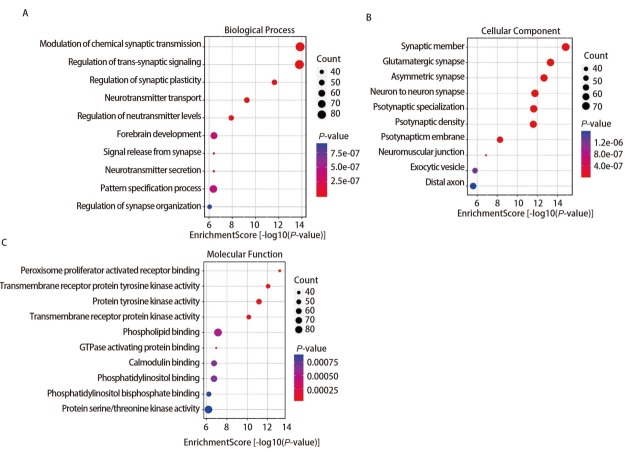
重叠靶基因的GO分析图。A：生物过程；B：细胞组分；C：分子功能。

**图 6:**
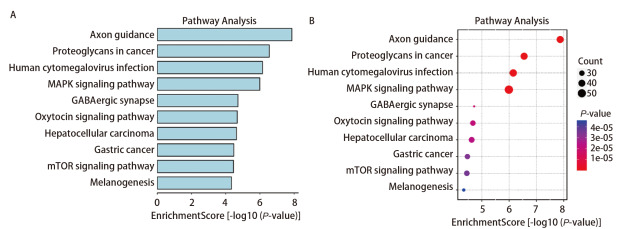
KEGG分析图。A：条形图；B：气泡图。

### 2.6 PPI网络构建及核心基因筛选

利用Cytoscape软件的CytoHubba插件筛选出20个核心基因，构建核心基因的PPI网络（图7A），其中miR-377-3p与3个核心基因相关（*EP300*、*AKT2*、*IGF1*）；miR-3960与0个核心基因相关；miR-4779与12个核心基因相关（*RPS6KB1*、*EGFR*、*GSK3B*、*SNAI1*、*STAT1*、*PIK3R1*、*RHOA*、*E2F1*、*CDKN1A*、*IGF1R*、*BCL2L11*、*CDC42*）；miR-6803-5p与8个核心基因相关（*AKT2*、*RELA*、*CDKN1A*、*KRAS*、*CREB1*、*IGF1*、*MDM2*、*TP53*）（图7B）。

**图 7:**
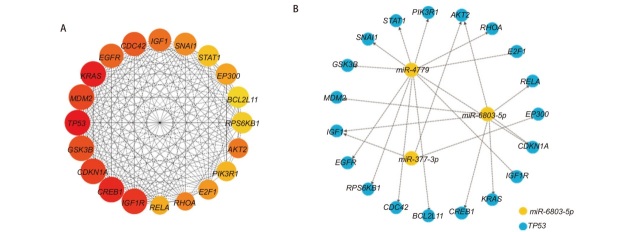
网络图。A：20个核心基因的PPI网络图；B：miRNA和核心基因关系图。

## 3 讨论

肺癌是全球大多数国家和地区癌症死亡的主要病因^[7]^。恶性肿瘤的早期检测可改善预后和生存率^[7]^，但大多数癌症发现时已处于晚期阶段，失去了手术治疗的机会，其预后通常较差。尽管组织活检是诊断肿瘤进展和转移的金标准^[7]^，但在癌症诊断中还需要更先进、更安全并且灵敏度和特异度高的方法来预测晚期LUSC的患者，为肺癌患者尽早提供手术时机，从而提高患者的OS，改善预后。

miRNAs与不同的细胞功能相关，控制着包括细胞增殖、分化和调控细胞死亡等在内的多种生物学过程^[8]^。miRNAs广泛参与基因表达和蛋白质的翻译环节，miRNAs通过调节癌基因和抑癌基因的表达，调控细胞增殖和细胞凋亡，从蛋白质、DNA和RNA水平上对肿瘤的发生、发展进行早期预警和预测，在肿瘤中发挥着潜在的作用^[9]^。另外，miRNAs作为一种理想的生物标志物，已经在乳腺癌、肺癌^[2,6]^、结直肠癌、黑色素瘤等恶性肿瘤中取得重要进展。本研究基于TCGA数据库进行数据分析，筛选可用于预测晚期LUSC预后的miRNAs标志物。并通过线上分析平台，构建miRNA-Gene网络，探索相关靶基因的机制，确定潜在的生物标志物，以提高诊断的准确性。

以往的相关研究中，针对miRNAs的研究已经取得了显著成效。例如，miR-let-7在肺癌细胞中下调*RAS*、*MYC*和*HMGA2*基因的表达^[10]^，进一步抑制*CDK6* mRNA表达，进而阻碍细胞周期的正常进程^[11]^。在NSCLC中，高表达miR-155和miR-let-7的患者预后较差^[12]^。而hsa-miR-126、hsa-miR-100、hsa-miR-145在肺癌中的表达水平较高，且与血管内皮生长因子（vascular endothelial growth factor, VEGF）家族呈负相关^[12]^。miR-146b可以用于预测LUSC的OS，miR-211-3p、miR-3679-3p和miR-4787-5p是不同阶段LUSC的理想标志物^[11]^。miR-1247-3p表达量的高低与肝细胞癌患者的肺转移相关^[13]^。Hu等^[14]^鉴定了含有miR-1、miR-30d、miR-486和miR-499的4种血清miRNAs特征，其预示了不同分期（I-IIIA期）NSCLC的OS。另有一些miRNAs也被鉴定为某种癌症的生物标志物，例如，胰腺癌中的hsa-miR-21-5p、hsa-miR-23a-3p和hsa-miR-27a-3p^[15]^以及淋巴瘤中的hsa-miR-142-3p和hsa-miR-494-3p。

本研究通过TCGA数据库，分析出7个miRNAs：miR-377-3p、miR-3960、miR-4779、miR-6803-5p、miR-1914-5p、miR-580-5p、miR-10a-5p，又根据ROC曲线分析每个miRNA的AUC值，最终确定了miR-377-3p、miR-3960、miR-4779、miR-6803-5p的AUC值具有统计学意义，且4个miRNAs的总体预测值为0.865，表明4个miRNAs的组合仍具有预测晚期LUSC的价值。

根据以往的研究，我们筛选的4个miRNAs参与了多种癌症及其他疾病的生物学过程，在疾病的诊断、预后分析及治疗中发挥重要作用。miR-377-3p对多种癌症的发生发展乃至预后均产生一定影响。高表达的miR-377-3p在TP53突变的患者中总体存活率较差^[16]^，miR-377的下调与肠型壶腹周围腺癌的最佳预后相关。Yuan等^[17]^的一项关于黑色素瘤的研究表明miR-377-3p可以抑制黑色素瘤的生长。Sun等^[18]^发现lncRNA NEAT1可以通过抑制miR-377-3p的作用促进NSCLC的进展。这些研究均证实miR-377-3p参与了多种癌症的调控过程，并对肿瘤的侵袭和转移表现出负向或正向调控。另有研究^[19]^发现，miR-3960可以抑制膀胱癌进展。miR-3960参与三阴性乳腺癌患者对顺铂的耐药性^[20]^。由此，我们可以发现miRNAs在不同类型的癌症或其他疾病，以及疾病的不同阶段，甚至治疗中产生了复杂的调控作用，具体机制仍需进一步探索。

miRNAs有助于维持调节细胞命运的基因之间的平衡，并且它们的失调可以破坏这种平衡，在癌症的起始到转移性疾病的发展或进展中均发挥一定的作用。由于miRNAs能够靶向多个分子，且1个靶基因或靶mRNA可以接受多个miRNAs的调控，这就产生了一个复杂的miRNA-mRNA网络，其中特定miRNAs的生物学效应和特性不是可以通过线性关系解释的。调节miRNAs表达的优点是它们能够同时靶向各种基因和途径^[8]^。为了进一步研究miRNAs的机制，本研究通过miRWalk和TargetScan预测miRNAs的靶基因并获取交集靶基因，通过STRING和Cytoscape等线上分析软件，最终筛选了20个核心基因，包括*CREB1*、*CDC42*、*TP53*、*KRAS*、*IGF1*、*IGF1R*、*CDKN1A*、*EGFR*、*SNAI1*、*GSK3B*、*AKT2*、*E2F1*、*RELA*、*EP300*、*PIK3R1*、*RPS6KB1*、*BCL2L11*、*STAT1*、*RHOA*、*MDM2*。

本研究筛选的20个核心基因在多种癌症的发生发展中发挥了重要的生物学功能。TP53是重要的抑癌基因，在癌症发展中起到关键作用，它参与细胞周期的调控、DNA损伤修复及细胞凋亡在内的多种生物学过程。CDC42是Rho家族的成员之一，它负责信号通路的启动，参与癌症的致病过程，CDC42的过表达干扰Ras和表皮生长因子受体（epidermal growth factor receptor, EGFR）的活性，诱导细胞转化，从而导致肿瘤的发生和发展^[21]^。AKT是一种STK，是PI3K途径的关键下游介质，活化的AKT在细胞存活、细胞周期进展和细胞生长调节中发挥重要作用^[22]^。AKT2是AKT家族的成员，已有研究^[22]^证明野生型AKT2的异位表达导致人乳腺癌和卵巢癌细胞的侵袭和转移，被认为是潜在的治疗靶点。KRAS属于原癌基因RAS家族，KRAS突变刺激其介导的信号转导途径，导致肿瘤的发生、进展、细胞过度增殖以及恶性发展和侵袭^[23]^。PI3K/PTEN信号通路的失衡被认为是多种肿瘤发生中的关键分子事件之一，PIK3R1则是PI3K的主要调节亚型，*PIK3R1*的突变作为肿瘤的抑制基因或癌基因影响疾病进展。*PIK3R1*的突变、缺失或表达水平异常可以引起PI3K/AKT/mTOR通路的活化，从而促进细胞生长、存活、增殖和迁移^[24]^。*MET*基因在多种癌症中发生异常改变时，常与癌细胞异常增殖及侵袭能力增强密切相关^[25]^。CREB1是一种转录因子，发挥致癌作用，参与肿瘤细胞的增殖、存活和转移。CREB1在乳腺癌组织和转移性癌细胞中升高，与预后不良、转移和淋巴结受累相关，并可作为乳腺癌的潜在标志物^[26]^，而CREB1在大多数NSCLC细胞系中高度表达，并与NSCLC患者的生存率降低相关。通过充分了解CREB1和miRNAs的调控网络，CREB1和miRNAs具有作为人类癌症治疗靶点的潜力。NSCLC中IGF-1和IGF1R的上调和表达与肿瘤进展和患者预后相关。一项关于LUSC的研究^[27]^显示，与其他类型的癌症相比，LUSC中的IGF1R蛋白高表达与EGFR表达相关，并且高*IGF1R*基因拷贝数是手术切除NSCLC的独立预后因素。EGFR是多种miRNAs的靶点，miRNAs参与肺癌发生和靶向治疗的EGFR信号通路，近来受到越来越多的关注，miRNAs通过靶向EGFR信号通路在不同类型的癌症中发挥作用。最近在LUSC中也发现了miR-206对c-MET和EGFR致癌信号通路的这种双重抑制作用^[28]^。研究证实几种miRNAs可以作为生物标志物来预测肺癌患者对EGFR-酪氨酸激酶抑制剂（EGFR-tyrosine kinase inhibitors, EGFR-TKIs）的反应^[29]^。另外，具有*EGFR*突变的根治性切除NSCLC患者比无*EGFR*突变NSCLC患者，miR-21和miR-10b均高表达^[30]^。另外，研究^[30]^证实越来越多的miRNAs与肺癌细胞对抗EGFR药物的耐药性相关，提示miRNAs可能成为抗EGFR药物治疗的新靶点，或将成为一项有前景的预测性生物标志物。这些研究表明，我们筛选miRNAs的核心靶基因在肿瘤的发展中起到重要作用，为后续关于miRNAs作用机制的探索提供了有价值的参考。

本研究基于数据库研究，对早期LUSC和晚期LUSC患者进行分析，得到一组miRNAs可以用来预测晚期LUSC的生物标志物，并对相关靶基因进行机制的探讨。基于先前针对miRNAs及其靶基因在各种癌症中的研究，我们认为本研究筛选出的miRNAs标志物可以用于晚期LUSC患者的早期诊断。但本研究的分析对象来自于数据库，并且尚未经过临床样本的分析验证，存在一定的局限性。且由于晚期LUSC样本数目的局限性，结果存在偏倚。此外，本研究根据miRNAs的表达量进行筛选，删除了表达量过低的miRNAs，减少了表达量过低的miRNAs对结果的干扰，但仍可能存在误差。另外多种混杂因素如人群、年龄和外部因素等都可以对最终结果产生影响，后续，我们会对临床样本进行严格的病例对照研究，进一步优化我们的miRNAs组合，并对其在生物学过程中的作用机制进行分析与讨论。

总之，肺癌由于其晚期确诊和治疗的有限性而具有高患病率和死亡率，miRNAs则是用于癌症的诊断和监测的一种理想标志物。另外，miRNAs稳定存在于患者的循环核酸中，具有组织特异性和高度稳定性，因此不仅作为诊断性生物标志物，甚至还可以作为治疗性和预后性生物标志物发挥着重要作用。虽然在miRNAs与癌症的相关性方面已经取得了许多进展，但进一步了解现有的形式和生物学功能，仍有许多难题需要进一步研究。
